# Osseous reaction to implantation of two endodontic cements: Mineral trioxide aggregate (MTA) and calcium enriched mixture (CEM)

**DOI:** 10.4317/medoral.18136

**Published:** 2012-05-01

**Authors:** Saeed Rahimi, Hadi Mokhtari, Shahriar Shahi, Ali Kazemi, Saeed Asgary, Mohammad J. Eghbal, Mehran Mesgariabbasi, Daryoush Mohajeri

**Affiliations:** 1 DDS, MSc, Professor of Endodontics, Dental and Periodontal Research Center, Department of Endodontics, Faculty of Dentistry, Tabriz University of Medical Sciences, Tabriz, Iran; 2 DDS, MSc, Assistant Professor, Dental and Periodontal Research Center, Department of Endodontics, Faculty of Dentistry, Tabriz University of Medical Sciences, Tabriz, Iran; 3 DDS, MSc, Assistant Professor, Department of Endodontics, Faculty of Dentistry, Shahed University of Medical Sciences, Tehran, Iran; 4 DDS, MSc, Professor of Endodontics, Iranian Center for Endodontic Research, Dental Research Center, Shahid Beheshti University M.C., Tehran, Iran; 5 DVM, Researcher, Drug Applied Research Center, Tabriz University of Medical Sciences, Tabriz, Iran; 6 PhD, Assistant Professor, Department of Pathobiology, Tabriz Branch, Islamic Azad University, Tabriz, Iran

## Abstract

Aim: The aim of the present in vivo study was to determine bone tissue reaction to calcium enriched mixture (CEM) and mineral trioxide aggregate (MTA) using a rat femur model. Study Design: Sixty-three rats were selected and randomly divided into three groups of 21 each [experimental groups (n=15), control (n=6)]. Implantation cavities were prepared in each femoral bone and randomly filled with the biomaterials only in the experimental groups. The animals in three groups were sacrificed 1, 4, and 8 weeks postoperatively. Histologic evaluations comprising inflammation severity and new bone formation were blindly made on H&E-stained decalcified 6-µm sections. Results: At 1, 4, and 8 weeks after implantation number of inflammatory cells had decreased in the CEM, MTA and control groups, respectively, with no statistically significant differences. Conversely, new bone formation had increased in all the experimental and control groups, without statistically significant differences. Conclusion: The results suggest that biocompatibility of MTA, as gold standard, and CEM cement as a new endodontic biomaterial are comparable

** Key words:**Endodontics, MTA,CEM, osseous reaction.

## Introduction

Mineral trioxide aggregate (MTA) has many of the characteristics of an ideal biomaterial for various endodontic treatments. MTA was introduced in 1993 as a root-end filling material (1). It is a mixture of calcium phosphate, calcium oxide, silicate and bismuth oxide (2). MTA exhibits proper biocompatibility in the proximity of pulp and periapical tissues (3-7); it is superior to other materials used for perforation repair and root-end filling (8). MTA has some disadvantages, including long setting time, weak handling properties, discoloration potential, high price and questionable antibacterial properties (9-11).

Recently, a new experimental cement (NEC) in the name of calcium-enriched mixture (CEM) cement (BioniqueDent, Tehran, Iran) consisting of different calcium compounds such as calcium oxide, calcium phosphate, calcium carbonate, calcium silicate, calcium sulfate, calcium hydroxide, and calcium chloride was developed (12).

CEM is a tooth-colored water-based cement with similar clinical applications as MTA, but with different chemical composition (12) and has exhibited proper sealing ability (9), antimicrobial properties similar to those of calcium hydroxide (13), hard tissue induction properties (14) and shorter setting time, greater flowability and lower film thickness compared to MTA (12).

Biocompatibility of materials is evaluated by various techniques, including ex vivo cytotoxicity and in vivo subcutaneous or intraosseous implantation procedures (15). Since no studies to date have evaluated the osseous reaction to CEM, the aim of the present in vivo study was to evaluate the bone tissue reaction of rat femur to CEM and compare it with those of MTA.

## Material and Methods 

Sixty-three mature healthy Dawley Wistar rats, weighing 250-300 grams, were selected. The rats were quarantined for 10 days after a veterinarian confirmed their health. The protocol of the study was reviewed and approved by the Ethics Committee of Tabriz Medical Sciences University. The animals were kept and treated according to recommendations of Helsinki Declaration.

The rats were randomly divided into three groups of 21. Each group consisted of an experimental subgroup (n=15) and a control subgroup (n=6). Each animal underwent a general anesthetic procedure by intraperitoneal injection of 10% Ketamine HCl (Alfasan, Woerden, the Netherlands) at a dose of 25 mg/kg body weight and Xylazine (Bayer, Munich, Germany) at a dose of 0.001 mL/kg body weight. The femur incision area was disinfected with 70% ethanol to gain access to rat femur. Incisions were made on two sides of access incisions in a sterile condition. Implantation cavities were prepared in each femoral bone with a diameter and depth of 1 mm, using a round carbide bur (D&Z, Wiesbaden, Germany) in a low-speed hand piece under normal saline irrigation. After irrigation with normal saline and control of bleeding, in the three experimental subgroups, ProRoot MTA (Dentsply, Tulsa Dental, OK, USA) was randomly used on one side and CEM cement (BioniqueDent, Tehran, Iran) was used on the other side. The materials were mixed according to manufacturer’s instructions and were directly placed in the osseous cavities. In the control subgroups the cavities were prepared in the same manner in femoral bones but no materials were placed in them. Therefore, in each control subgroup, 12 control samples were provided. The incisions were then closed with 4-0 silk sutures and the animals were subjected to the same diet and environmental conditions.

The animals in groups 1, 2, and 3 were sacrificed 1, 4, and 8 weeks after surgery, respectively, by putting them in a carbon dioxide chamber for 5-10 minutes. Subsequently, the animals` femurs were removed and placed in 10% buffered formalin. The bones were embedded in paraffin after decalcification in 10% formic acid. Then 6-µm serial sections were prepared and stained with hematoxylin-eosin (H&E). The specimens were evaluated in a blind manner under a light microscope (Zeiss, Goettingen, Germany) by a pathologist blind to the procedure. The inflammation in the area was determined by counting the inflammatory cells, including lymphocytes, plasma cells and macrophages at ×400. The inflammation severity was graded based on inflammatory cell counts according to studies carried out by Noetzel et al. (16) and Panzarini et al. (17) as follows:

- Grade 0: No inflammatory cells.

- Grade I: Inflammatory cells < 25

- Grade II: Inflammatory cells = 25-50

- Grade III: Inflammatory cells = 51-75

- Grade IV: Inflammatory cells > 75

Evaluation of new bone formation around the implanted materials was carried out at ×40 or ×100 as follows (18):

- Grade 0: No bone formation.

- Grade I: Slight; presence of bony islets and coverage of less than 25% of the material surface with bone.

- Grade II: Moderate; coverage of at least 50% of the material surface with bone.

- Grade III: Extensive; complete coverage of the material surface with bone or the formation of an osseous bridge around the material.

Data were collected and analyzed with descriptive statistical methods (frequency; percentage) and chi-squared test. Statistical significance was defined at P < 0.05.

## Results

In the present study the severity of inflammatory processes and the extent of bone formation adjacent to the biomaterials were evaluated at 1-, 4-, and 8-week intervals. There were no adverse inflammatory foreign-body reactions adjacent to the two experimental biomaterials.

Evaluation of inflammation

1-week-old specimens 

**Figure 1:**
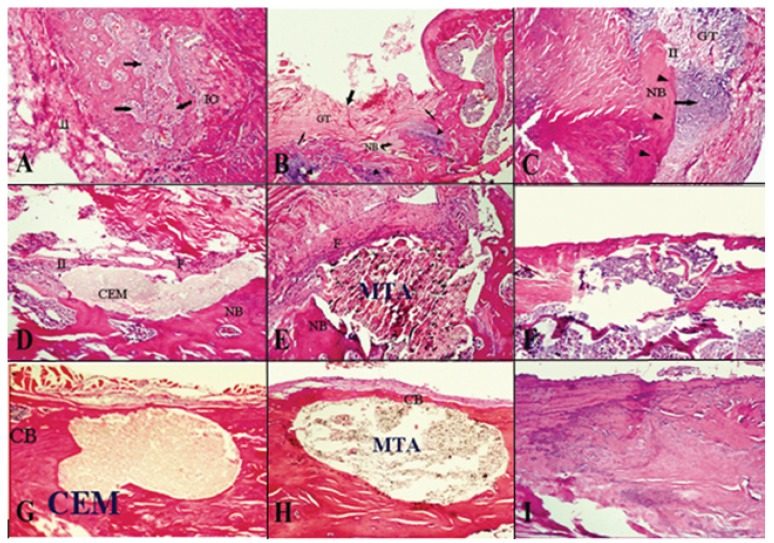
Histologic images of inflammatory cell infiltration and bone formation around experimental and control groups (hematoxylin and eosin staining). (A) 1-week-old CEM specimens; (original magnification, x100). (B) 1-week-old MTA specimens; (original magnification, x40). (C ) 1-week-old control specimens; (original magnification, x100). (D) 4-week-old CEM specimens; (original magnification, new bone tissue formation; F: fibrosis tissue; CB: complete bridge bone formation).x100). (E) 4-week-old MTA specimens; (original magnification, x100). (F) 4-week-old control specimens; (original magnification, x40). (G) 8-week-old CEM specimens; (original magnification, x100). (H) 8-week-old MTA specimens; (original magnification, x100). (I) 8-week-old control specimens; (original magnification, x100). (IO: Intraosseous bone formation; II: inflammatory cell infiltration; GT: granulation tissue; NB: new bone formation; CB: complete bone bridge formation; F: fibrosis tissue).

Severe infiltration of inflammatory cells, including lymphocytes, plasma cells and macrophages, was observed in the two experimental and control groups. The inflammatory processes were graded as III or IV, with no significant differences between the groups (P = 0.71) ( Table 1, Fig. 1).

**Table 1:**
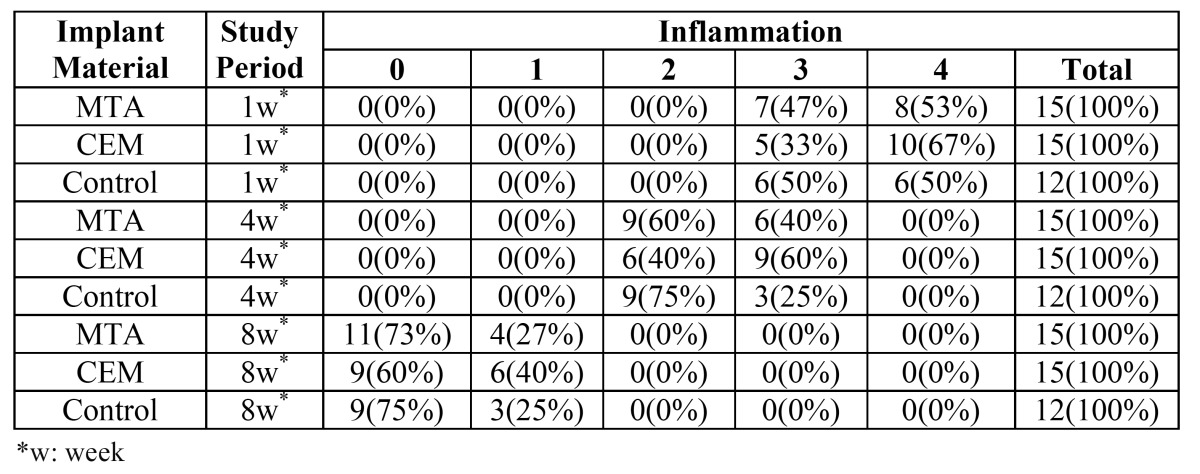
Inflammatory grades adjacent to MTA, CEM and in the controls at 1-, 4-, and 8-week intervals.

4-week-old specimens 

The number of inflammatory cells had decreased in all the three groups and the inflammatory processes were graded as II or III, with no statistically significant differences between the groups (P = 0.46) ( Table 1, Fig. 1).

8-week-old specimens 

Lymphocyte, plasma cell and macrophage counts had significantly decreased in the three groups. The inflammatory processes were graded as I or 0, with no statistically significant differences between the three groups (P = 0.63) ( Table 1, Fig. 1).

Evaluation of bone formation

The results of the evaluation of new bone formation processes around the experimental biomaterials at 1-, 4-, and 8-week intervals are summarized in ( Table 2). Bone formation processes were similar in the CEM, MTA and control groups, with no statistically significant differences (P = 0.54) ( Table 2).

**Table 2:**
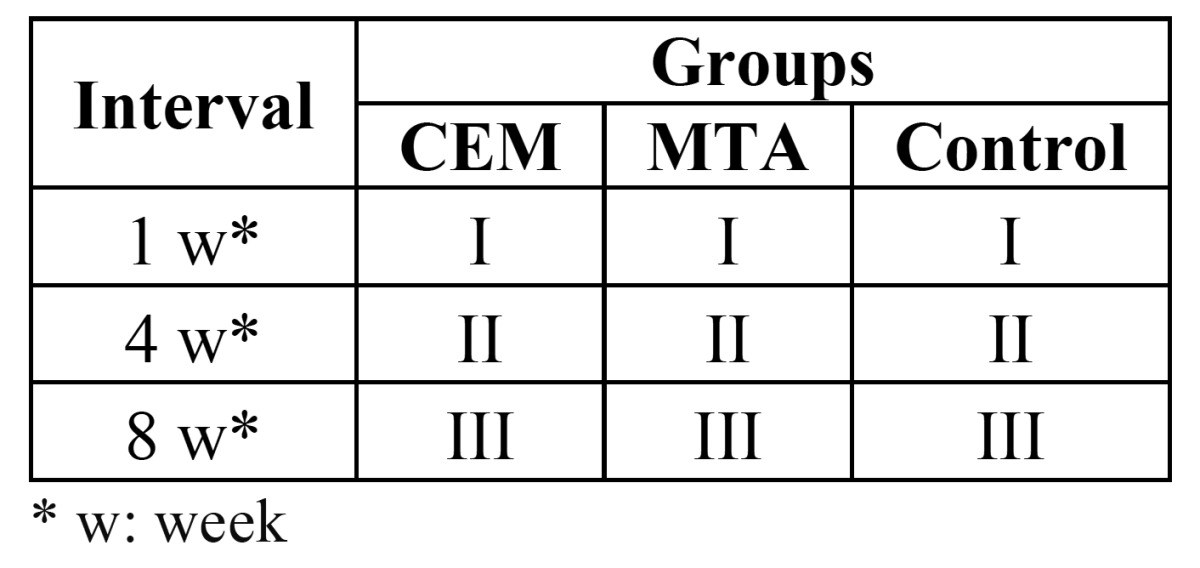
Bone formation data around the biomaterials under study in the control group.

## Discussion

Endodontic materials frequently come in close contact with soft and hard tissues of the periodontium. Therefore, it is necessary for an endodontic material to be biocompatible, which is especially important for root-end filling materials such as MTA and CEM cement.

In the majority of studies carried out on the tissue reaction to materials implanted in bone the duration of experiments have been thirty (19), sixty (18) or ninety (20) days. The present study lasted 8 weeks, which is consistent with the majority of studies in this respect (18,19).

A metaanalysis is made of filler materials in periapical surgery, evaluating a total of 30 articles published in recent years, have shown that MTA appears to be an ideal material (21), which is consistent with the results of the present study.

Although the chemical composition of CEM is different from that of MTA (12), they have similar clinical applications. When CEM is used for pulp capping (14) or as a root-end filling material (22) results have been similar to those achieved with MTA. In addition, CEM has yielded appropriate results when used in revascularization of necrotic molars (23), and in the management of external root resorption (24). A recent study compared the tissue response to CEM and MTA in the treatment of furcal perforations in the teeth of dogs and showed that MTA and CEM have similar and proper tissue responses to the formation of cement um-like hard tissues. Furthermore, inflammatory processes and osseous bridge formation have been similar in the vicinity of both materials (25). The results of previous studies are consistent with those of the present study in relation to similarities of properties of the two materials.

Regarding biocompatibility of MTA, its chemical composition and the related reactions should be taken into account. MTA produces a substance similar to hydroxyapatite in the presence of synthetic tissue fluid (STF). In a study (26) it was concluded that MTA is catalyzed in the presence of tissue fluids and releases all its cationic content, of which calcium has the highest proportion. As a result of MTA porosity, it seems this reaction occurs inside the material, too, changing the composition of MTA in the vicinity of tissues. This hydroxyapatite layer is highly biocompatible, with low toxicity. This layer might have osteogenic potential because it can release calcium and phosphorus ions, which are involved in bone metabolism. According to the results of another study hydroxyapatite formation has been observed adjacent to MTA, which has been reported to have a role in its biocompatibility (27).

CEM is composed of various calcium components, which provide a rich reservoir of calcium and phosphorus ions. These elements have a role in the process of hydroxyapatite formation, which is a natural product of dental pulp cells (28). This property, similar to the reaction explained in the case of MTA, might have a role in the biocompatibility of CEM. Scanning electron microscopic studies have shown that the distribution pattern of calcium, phosphorus and oxygen in the CEM as a root-end filling material is similar to that of surrounding dentin (29).

In the present study the inflammatory process during the first week in the MTA and CEM groups was of grades 3 or 4; however, the inflammation grade decreased to 1 or 0 in both groups at the end of the eighth week, which is consistent with the results of previous studies (4,7,18,25). The higher inflammation grades in the first week might be attributed to various factors, including high pH value, production of heat during the setting reaction and the release of IL 1 and IL 6 (30). In addition, the trauma as a result of surgery should be considered because in the control groups the inflammatory process during the first week was of grade 2 or 3.

Another variable evaluated in the present study was bone formation, with similar results in the three groups. One week after implantation grade I, four weeks later grade II, and eight weeks later grade III bone formations were evident, which is consistent with the results of other studies (18). This process might be attributed to calcium-containing components in both MTA and CEM cements. As it was previously explained calcium hydroxide is produced as a result of hydration reaction; if calcium hydroxide is leached out it might induce calcified bridge formation (30).

Based on the results of the present in vivo study it was concluded that biocompatibility of MTA and formation of osseous tissues in its vicinity, as gold standard, and CEM as a new endodontic biomaterial are similar and, on the whole, satisfactory. CEM has biologic properties similar to those of MTA and can be used in endodontic procedures due to its proper physical properties and easy handling. However, further studies are deemed necessary to substantiate its clinical efficacy.
